# When the path is blocked: A case of a transverse vaginal septum diagnosed in labor

**DOI:** 10.1002/ijgo.70626

**Published:** 2025-11-14

**Authors:** Beatrice Lynch, Lama Noureddine, Samatha Irizarry, Shauna Williams, Chavi Eve Karkowsky

**Affiliations:** ^1^ Department of Obstetrics, Gynecology, and Reproductive Health Rutgers New Jersey Medical School Newark New Jersey USA

**Keywords:** case report, intrapartum diagnosis, Müllerian anomalies, point of care ultrasound, septum recurrence, transverse vaginal septum

## Abstract

Transverse vaginal septa are rare, with incidence estimated to be between 1 in 30 000 to 1 in 84 000 people. Despite much literature in the gynecologic setting, there is little guidance about how to manage them when diagnosed in labor. A 23‐year‐old G1P000 patient presented to Labor and Delivery at 40 weeks 0 days with contractions and vaginal bleeding. A sterile bimanual exam found a blind vaginal ending, with no identifiable cervix. A point of care transvaginal ultrasound showed a transverse septum that measured 0.39 cm in thickness, with a normal cervix behind the septum. The patient was offered an exam under anesthesia with possible septum resection or primary cesarean delivery. She elected for an exam under anesthesia, which revealed a small area of dimpling on the septum. This was bluntly dissected along the scar of the previous resection, without hemorrhage or other complications. Subsequently, the cervix was identified, and the exam was 5 cm dilated, 80% effaced. The patient progressed to an uncomplicated vaginal delivery with no hemorrhage noted in the antepartum or postpartum period. Though there is no consensus or official recommendation for how to manage transverse vaginal septa during labor, there is a concern that a transverse vaginal septum during labor can lead to bleeding or obstructed labor, which can potentially result in uterine rupture. Our patient was diagnosed via transvaginal ultrasound, allowing for blunt dissection and ultimately an uncomplicated vaginal delivery; and this option should be considered for such patients identified in the intrapartum period.

A 23‐year‐old, G1P0 patient presented to Labor and Delivery at 40 weeks 0 days of pregnancy with a complaint of contractions. She had received routine prenatal care initiated at 21 weeks 1 day. The speculum examination performed at that time had shown a cervix with normal appearance. A cervical smear showed high‐grade squamous intraepithelial lesion and was HPV negative. Colposcopy attempted at 33 weeks was unable to be completed due to pain. The patient did not have any other cervical examinations before presenting to Labor and Delivery. She became pregnant without the use of reproductive technology but reported having a “surgery so she could have periods” in Ecuador at age 12 years. She was not sure of the exact nature of the surgery, and no operative report was available. At Labor and Delivery, a speculum could not be inserted, and a sterile bimanual examination found a blind vaginal ending. Transvaginal ultrasound examination showed a transverse septum that measured 0.39 cm in thickness (Figure 1).

**FIGURE 1:**
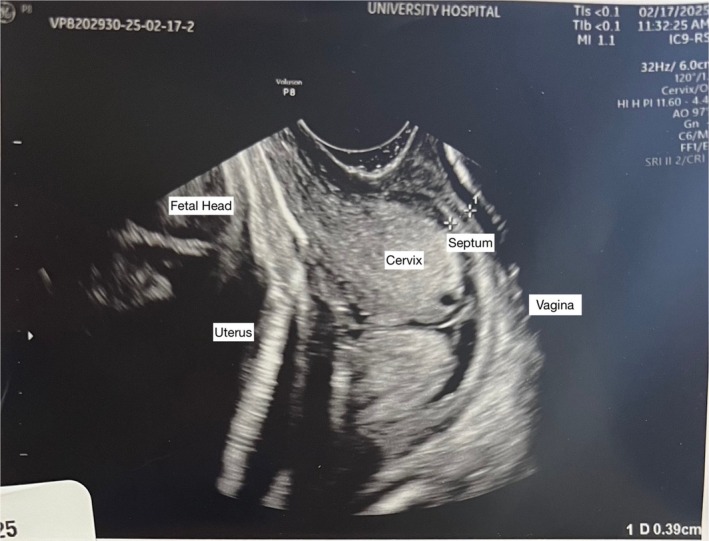
Annotated point‐of‐care transvaginal ultrasound image demonstrating transverse vaginal septum at time of diagnosis.

The patient was offered an examination under anesthesia with possible septum resection or a primary cesarean section, which she elected to undergo. This revealed a small area of dimpling. This was bluntly dissected along the scar of the previous resection with minimal blood loss. Cervical examination was subsequently found to be 5 cm dilated, 80% effaced, and ‐3 station.  Fetal heart tracing remained category I throughout the course of labor, and the patient had a spontaneous vaginal delivery. Uterine atony was noted after delivery of the placenta, and she received methylergonovine 0.2 mg intramuscularly. Total estimated blood loss was 500 mL. The site of the vaginal septal resection was visualized after delivery, and was hemostatic with pressure. The patient was noted as normal and was discharged on day 2 postpartum. Consent from this patient to write this case report was obtained via telephone on May 21, 2025. This case report was deemed to not require approval from an ethics committee.

Transverse vaginal septa are rare, with an incidence between 1 in 30 000 and 1 in 84 000 women.^1^ This patient likely had an incomplete transverse septum, which may have been corrected at the time of her surgery at age 12 years, with possible regrowth during pregnancy. The rate of recurrence is thought to be between 7% and 21%,^2^ with an increase in pregnancy. The increased estrogen levels and vascularity of pregnancy promote tissue proliferation, and the mechanical pressure from the enlarging uterus may contribute to re‐adhesion of the vaginal walls.^3^

There is conflicting guidance on the intrapartum management of patients with a transverse vaginal septum. There is a concern that a septum can lead to obstructed labor and uterine rupture,^4^ so some medical workers would always recommend a cesarean delivery. Others have proposed expectant management with spontaneous dissection of the septum as the fetus descends, or incision after the septum has been thinned and there is pressure from the fetus for hemostasis.^5^ One study concluded that incision before labor was not optimal because it can lead to scarring, which can result in obstructed labor.^6^ However, a case report of two patients with transverse vaginal septa who were allowed a trial of labor with septal incision during active labor, reported that they had vaginal deliveries with no related complications.^7^

In conclusion, although there are no clear guidelines, this case shows that blunt dissection under anesthesia with ultimate progress of labor can be considered when managing a woman with a transverse vaginal septum in labor. Further research is required, however, to create true guidelines. The scope of future research could include a study following patients who had septum resection during their pregnancy; comparing their obstetrical outcomes as well as the predictability of obstetrical complications using artificial intelligence based on sonographic appearance of septa.

## AUTHOR CONTRIBUTIONS

BL contributed to the conception and design of the case report, drafted the initial manuscript, and participated in revisions. LN, SI, SW, and CEK contributed to the conception and design of the case report and participated in revisions.

## FUNDING INFORMATION

None received.

## CONFLICT OF INTEREST STATEMENT

The authors have no conflicts of interest.

## Data Availability

Data sharing is not applicable to this article as no new data were created or analyzed in this study.
